# Automated collateral assessment restricted to the hypoperfused area for distal vessel occlusions in ischemic stroke

**DOI:** 10.1007/s00330-025-11442-2

**Published:** 2025-04-14

**Authors:** Lucas de Vries, M. M. Quirien Robbe, Ivo G. H. Jansen, S. Mahsa Mojtahedi, Jan W. Hoving, Susanne G. H. Olthuis, Robrecht R. M. M. Knapen, Florentina M. E. Pinckaers, Manon Kappelhof, Ludo F. M. Beenen, Alida A. Postma, Robert J. van Oostenbrugge, Diederik W. J. Dippel, Efstratios Gavves, Bart J. Emmer, Charles B. L. M. Majoie, Wim H. van Zwam, Henk A. Marquering, on behalf of the MR CLEAN Registry Investigators, Diederik W. J. Dippel, Aad van der Lugt, Charles B. L. M. Majoie, Yvo B. W. E. M. Roos, Robert J. van Oostenbrugge, Wim H. van Zwam, Jelis Boiten, Jan Albert Vos, Ivo G. H. Jansen, Maxim J. H. L. Mulder, Robert-Jan B. Goldhoorn, Kars C. J. Compagne, Manon Kappelhof, Josje Brouwer, Sanne J. den Hartog, Wouter H. Hinsenveld, Lotte van den Heuvel, Diederik W. J. Dippel, Aad van der Lugt, Charles B. L. M. Majoie, Yvo B. W. E. M. Roos, Robert J. van Oostenbrugge, Wim H. van Zwam, Jelis Boiten, Jan Albert Vos, Bob Roozenbeek, Pieter Jan van Doormaal, Bart J. Emmer, Jonathan M. Coutinho, Wouter J. Schonewille, Marieke J. H. Wermer, Marianne A. A. van Walderveen, Adriaan C. G. M. van Es, Julie Staals, Jeannette Hofmeijer, Jasper M. Martens, Geert J. Lycklama à Nijeholt, Sebastiaan F. de Bruijn, Lukas C. van Dijk, H. Bart van der Worp, Rob H. Lo, Ewoud J. van Dijk, Hieronymus D. Boogaarts, J. de Vries, Paul L. M. de Kort, Julia van Tuijl, Issam Boukrab, Jo P. Peluso, Puck Fransen, Jan S. P. van den Berg, Heleen M. den Hertog, Boudewijn A. A. M. van Hasselt, Leo A. M. Aerden, René J. Dallinga, Maarten Uyttenboogaart, Omid Eschgi, Reinoud P. H. Bokkers, Tobien H. C. M. L. Schreuder, Roel J. J. Heijboer, Koos Keizer, Rob A. R. Gons, Lonneke S. F. Yo, Emiel J. C. Sturm, Tomas Bulut, Paul J. A. M. Brouwers, Anouk D. Rozeman, Otto Elgersma, Michel J. M. Remmers, Thijs E. A. M. de Jong, Aad van der Lugt, Charles B. L. M. Majoie, Wim H. van Zwam, Jan Albert Vos, Bart J. Emmer, Marianne A. A. van Walderveen, Adriaan C. G. M. van Es, Jasper M. Martens, Geert J. Lycklama à Nijeholt, Hieronymus D. Boogaarts, Jo P. Peluso, Maarten Uyttenboogaart, Reinoud P. H. Bokkers, Lonneke S. F. Yo, Marieke E. S. Sprengers, Sjoerd F. M. Jenniskens, René van den Berg, Albert J. Yoo, Ludo F. M. Beenen, Alida A. Postma, Stefan D. Roosendaal, Bas F. W. van der Kallen, Ido R. van den Wijngaard, Joost Bot, Pieter-Jan van Doormaal, Anton Meijer, Elyas Ghariq, Marc P. van Proosdij, G. Menno Krietemeijer, Rob Lo, Wouter Dinkelaar, Auke P. A. Appelman, Bas Hammer, Sjoert Pegge, Anouk van der Hoorn, Saman Vinke, Sandra Cornelissen, Christiaan van der Leij, Rutger Brans, Jeanette Bakker, Miou Koopman, Lucas Smagge, Olvert A. Berkhemer, Jeroen Markenstein, Eef Hendriks, Patrick Brouwer, Dick Gerrits, Diederik W. J. Dippel, Aad van der Lugt, Charles B. L. M. Majoie, Yvo B. W. E. M. Roos, Robert J. van Oostenbrugge, Wim H. van Zwam, Jelis Boiten, Jan Albert Vos, Wouter J. Schonewille, Jeannette Hofmeijer, Jasper M. Martens, Geert J. Lycklama à Nijeholt, H. Bart van der Worp, Rob H. Lo, Robert J. van Oostenbrugge, Jeannette Hofmeijer, H. Zwenneke Flach, Hester F. Lingsma, Naziha el Ghannouti, Martin Sterrenberg, Wilma Pellikaan, Rita Sprengers, Marjan Elfrink, Michelle Simons, Marjolein Vossers, Joke de Meris, Tamara Vermeulen, Annet Geerlings, Gina van Vemde, Tiny Simons, Gert Messchendorp, Nynke Nicolaij, Hester Bongenaar, Karin Bodde, Sandra Kleijn, Jasmijn Lodico, Hanneke Droste, Maureen Wollaert, Sabrina Verheesen, D. Jeurrissen, Erna Bos, Yvonne Drabbe, Michelle Sandiman, Nicoline Aaldering, Berber Zweedijk, Jocova Vervoort, Eva Ponjee, Sharon Romviel, Karin Kanselaar, Denn Barning, Laurine van der Steen, Olvert A. Berkhemer, Esmee Venema, Vicky Chalos, Ralph R. Geuskens, Tim van Straaten, Saliha Ergezen, Roger R. M. Harmsma, Daan Muijres, Anouk de Jong, Anna M. M. Boers, J. Huguet, P. F. C. Groot, Marieke A. Mens, Katinka R. van Kranendonk, Kilian M. Treurniet, Manon L. Tolhuisen, Heitor Alves, Annick J. Weterings, Eleonora L. F. Kirkels, Eva J. H. F. Voogd, Lieve M. Schupp, Sabine L. Collette, Adrien E. D. Groot, Natalie E. LeCouffe, Praneeta R. Konduri, Haryadi Prasetya, Nerea Arrarte-Terreros, Lucas A. Ramos, Nikki Boodt, Anne F. A. V. Pirson, Agnetha A. E. Bruggeman, Nadinda A. M. van der Ende, Rabia Deniz, Susanne G. H. Olthuis, Floor Pinckaers

**Affiliations:** 1 Biomedical Engineering and Physics, Amsterdam UMC location, University of Amsterdam, Amsterdam, The Netherlands; 2 Radiology and Nuclear Medicine, Amsterdam UMC location, University of Amsterdam, Amsterdam, The Netherlands; 3 Informatics Institute, University of Amsterdam, Amsterdam, The Netherlands; 4 Department of Radiology and Nuclear Medicine, Maastricht University Medical Center, Maastricht, The Netherlands; 5 Cardiovascular Research Institute Maastricht (CARIM), Maastricht University, Maastricht, The Netherlands; 6 Nicolab B.V., Amsterdam, The Netherlands; 7 Department of Neurology, Maastricht University Medical Center, Maastricht, The Netherlands; 8 Department of Radiology and Nuclear Medicine, Maastricht University Medical Center+, Maastricht, The Netherlands; 9 School for Cardiovascular Diseases (CARIM), Maastricht University, Maastricht, The Netherlands; 10 School for Mental Health and Scienses (Mhens), Maastricht University, Maastricht, The Netherlands; 11 Neurology, Erasmus University Medical Center, Rotterdam, The Netherlands; 12 Department of Neurology, Erasmus MC University Medical Center, Rotterdam, The Netherlands; 13 Radiology, Erasmus MC University Medical Center, Rotterdam, The Netherlands; 14 Department of Radiology and Nuclear Medicine, Amsterdam UMC location, University of Amsterdam, Amsterdam, The Netherlands; 15 Neurology, Amsterdam UMC location, University of Amsterdam, Amsterdam, The Netherlands; 16 Department of Neurology, Maastricht University Medical Center+, Maastricht, The Netherlands; 17 School for Cardiovascular Diseases Maastricht (CARIM), Maastricht, The Netherlands; 18 Radiology & Nuclear Medicine, Maastricht University Medical Center+, Maastricht, The Netherlands; 19 Neurology, Haaglanden MC, The Hague, The Netherlands; 20 Radiology, Sint Antonius Hospital, Nieuwegein, The Netherlands; 21 Public Health, Erasmus MC University Medical Center, Rotterdam, The Netherlands; 22 Department of Neurology, Sint Antonius Hospital, Nieuwegein, The Netherlands; 23 Department of Neurology, Leiden University Medical Center, Leiden, The Netherlands; 24 Radiology, Leiden University Medical Center, Leiden, The Netherlands; 25 Department of Neurology, Rijnstate Hospital, Arnhem, The Netherlands; 26 Radiology, Rijnstate Hospital, Arnhem, The Netherlands; 27 Department of Radiology, Haaglanden MC, The Hague, The Netherlands; 28 Department of Neurology, HAGA Hospital, The Hague, The Netherlands; 29 Radiology, HAGA Hospital, The Hague, The Netherlands; 30 Department of Neurology, University Medical Center Utrecht, Utrecht, The Netherlands; 31 Radiology, University Medical Center Utrecht, Utrecht, The Netherlands; 32 Department of Neurology, Radboud University Medical Center, Nijmegen, The Netherlands; 33 Neurosurgery, Radboud University Medical Center, Nijmegen, The Netherlands; 34 Department of Neurology, Isala Klinieken, Zwolle, The Netherlands; 35 Department of Neurology, Elisabeth-TweeSteden ziekenhuis, Tilburg, The Netherlands; 36 Radiology, Elisabeth-TweeSteden ziekenhuis, Tilburg, The Netherlands; 37 Radiology, Isala Klinieken, Zwolle, The Netherlands; 38 Department of Neurology, Reinier de Graaf Gasthuis, Delft, The Netherlands; 39 Radiology, Reinier de Graaf Gasthuis, Delft, The Netherlands; 40 Department of Neurology, University Medical Center Groningen, Groningen, The Netherlands; 41 Radiology, University Medical Center Groningen, Groningen, The Netherlands; 42 Department of Neurology, Zuyderland Medical Center, Heerlen, The Netherlands; 43 Radiology, Zuyderland Medical Center, Heerlen, The Netherlands; 44 Department of Neurology, Catharina Hospital, Eindhoven, The Netherlands; 45 Radiology, Catharina Hospital, Eindhoven, The Netherlands; 46 Radiology, Medisch Spectrum Twente, Enschede, The Netherlands; 47 Department of Neurology, Medisch Spectrum Twente, Enschede, The Netherlands; 48 Department of Neurology, Albert Schweitzer Hospital, Dordrecht, The Netherlands; 49 Radiology, Albert Schweitzer Hospital, Dordrecht, The Netherlands; 50 Department of Neurology, Amphia Hospital, Breda, The Netherlands; 51 Radiology, Amphia Hospital, Breda, The Netherlands; 52 Radiology, Radboud University Medical Center, Nijmegen, The Netherlands; 53 Department of Radiology, Texas Stroke Institute, Plano, TX USA; 54 MHeNs School for Mental Health and Neuroscience, Maastricht, The Netherlands; 55 Department of Radiology, Amsterdam UMC, Vrije Universiteit van Amsterdam, Amsterdam, The Netherlands; 56 Department of Radiology, Noordwest Ziekenhuisgroep, Alkmaar, The Netherlands; 57 Biomedical Engineering & Physics, Amsterdam UMC location, University of Amsterdam, Amsterdam, The Netherlands; 58 Present Address: Deventer Hospital, Deventer, The Netherlands

**Keywords:** Stroke, Brain, Computed tomography angiography, Perfusion imaging, Collateral circulation

## Abstract

**Objectives:**

This study aims to: (1) develop and evaluate a quantitative assessment of collateral status in the downstream area of an occluded intracranial artery in acute ischemic stroke and compare this method to middle cerebral artery (MCA)-based assessment; (2) determine the agreement between the automated occlusion-downstream area collateral score (ODACS) and expert raters’ assessments, and compare this to inter-rater agreement.

**Methods:**

Patients from MR CLEAN-NO IV and MR CLEAN Registry with a proximal M1, distal M1, or M2 occlusion were included. Using the hypoperfused area from CT perfusion (CTP) as a proxy for the occlusion-downstream territory and automated vessel segmentations from CT angiography (CTA), ODACS is calculated as the vessel volume ratio between downstream ipsilateral and its contralateral regions. ODACS was compared to a whole MCA-territory approach and evaluated against visual scoring by two expert raters that visually estimated ODACS using CTA and CTP, and their inter-rater agreement.

**Results:**

The study included 204 patients with a proximal M1 (52%), distal M1 (32%), or M2 (16%) occlusion. ODACS yielded lower collateral scores than MCA-based scoring for all occlusion locations, with larger differences in more distal occlusions. For M2 occlusions, 58% of patients shifted from good (> 50%) to poor (≤ 50%) collateral filling of the occluded territory using ODACS. Moderate (weighted Cohen’s kappa κ = 0.45) inter-rater agreement and fair (κ = 0.35) to moderate (κ = 0.51) ODACS-rater agreement were observed.

**Conclusions:**

ODACS yields lower collateral scores compared to MCA-based scoring and is comparable to scores from expert raters.

**Key Points:**

***Question*** *CT angiography-based collateral assessment in the MCA territory is inadequate to assess the collateral status in patients with distal vessel occlusions*.

***Findings*** *Our automated ODACS revealed lower collateral scores than traditional whole-territory assessment, especially in distal vessel occlusions*.

***Clinical relevance*** *The more precise evaluation of affected brain territories through automated occlusion-downstream area assessments prevents an overestimation of collateral status in distal occlusions, which could lead to improved patient selection and treatment decisions in acute stroke care*.

**Graphical Abstract:**

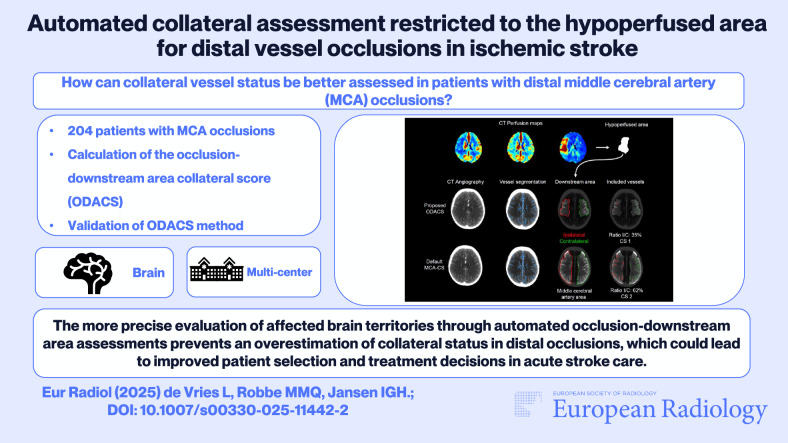

## Introduction

The presence of a patent collateral artery network, or in short *collaterals*, is a prognostic factor associated with good functional outcome in acute ischemic stroke patients [1]. Moreover, collaterals modify the treatment effect for endovascular treatment (EVT) in the early window, with a larger treatment effect in patients with better collaterals [2]. To some extent, collaterals can maintain perfusion to the affected brain tissue and slow down the progression from tissue at risk (penumbra) to infarcted tissue (core). Various (automatic) collateral scores (CSs) have been proposed to assess collateral status [1, 3–8]. Such scores commonly assess the middle cerebral artery (MCA) downstream territory filling, as they were developed for proximal large vessel occlusions [3, 8–10]. For proximal occlusions, where the (near-)whole MCA-territory is involved, this represents a straightforward, efficient, fast, and pragmatic approach [9–13]. However, in distal occlusions, assessing the whole MCA-territory will overestimate the collateral status since vessels in the unaffected territory are incorrectly counted as collateral flow - which in turn may reduce the predictive value of the CS. Given the advancement in EVT devices and experience, there is an emerging trend to treat more patients with distal occlusions [14–19]. To evaluate the effect of collaterals on treatment benefits in patients with distal occlusions, a novel approach for downstream collateral assessment is needed.

We propose an automated occlusion-downstream area collateral score (ODACS) to address these limitations. This novel method provides an objective assessment of collateral circulation specifically for the affected territory in patients with distal occlusions, overcoming both the overestimation issues of MCA-territory methods and challenges of visual assessment due to variations in cerebral vessel anatomy of more distal vessels [20–22]. Our primary objective is to develop a method to accurately determine the collateral status of patients with more distal occlusions without requiring explicit localization of the thrombus and which is robust to variations in vessel anatomy. To achieve this, we introduce and investigate an approach to collateral scoring using a patient-specific occlusion-downstream area that we obtain from the computed tomography (CT) perfusion-based hypoperfused region. Since vessels that are not part of the occlusion-downstream area are disregarded in the proposed methodology, we hypothesize that this approach will result in lower CSs for patients with distal occlusions compared to scores assessing the complete MCA-territory.

## Methods

### Data sets

We used data from a European multi-center, prospective, randomized clinical trial, MR CLEAN-NO IV [23], and from a Dutch prospective, nationwide, observational cohort study, the MR CLEAN Registry [24]. The studies included patients with large vessel occlusion acute ischemic stroke between March 2014 and October 2020. The medical ethics committee of the Erasmus MC University Medical Center, Rotterdam, the Netherlands, evaluated the protocols and granted permission for the MR CLEAN-NO IV trial (MEC-2017-368) and MR CLEAN Registry cohort study (MEC-2014-235). The ethics committee waived the necessity of written informed consent for the MR CLEAN Registry; written informed consent was obtained for all patients in MR CLEAN-NO IV. Details about in- and exclusion criteria were described elsewhere [23, 24]. From these studies, we retrospectively included a subset of patients. Specifically, we included patients who underwent computed tomography angiography (CTA) and computed tomography perfusion (CTP) at baseline, and of whom the CTP scans were processed with the software StrokeViewer (Nicolab; www.nicolab.com/strokeviewer) as part of the CLEOPATRA health care evaluation study [25]. The CLEOPATRA healthcare evaluation study was performed to assess the costs and health effects of CTP for selection for EVT. In CLEOPATRA, all available CTPs in the MR CLEAN Registry and MR CLEAN-NO IV were processed with CTP software from multiple vendors if possible. Not all CTPs could be processed, for example, due to missing source data, suboptimal quality, or faulty anonymization that led to corrupted source data. The resulting database had 169 patients with StrokeViewer CTP outcomes for the MR CLEAN Registry and 176 for MR CLEAN-NO IV. We used a single software to ensure consistent CTP outcomes. The CTP perfusion maps, penumbra and core masks, and baseline image reconstructions had 5 mm slice thickness. No standardized injection protocols were used. We excluded patients with CTA slice thickness exceeding 2 mm. We visually assessed the CTA phase and excluded patients with venous contrast enhancement following a similar procedure as published previously [26]. Since an ICA(-T) occlusion could introduce hypoperfusion beyond the MCA-territory, we excluded patients with such occlusions.

### Occlusion-downstream area CS

Our proposed automated ODACS aims to quantify collateral circulation in the patient-specific occlusion-downstream area that we obtain from the CTP-based hypoperfused region. We describe the steps to calculate the ODACS in the following paragraphs.

#### Alignment and co-registration

We used a midline plane extracted by StrokeViewer to align and center the midline plane of the CTP scans with a standard coordinate system. We applied the same alignment to the CTP outcomes and co-registered the CTA to the first frame of the CTP sequence.

#### Vessel segmentation

We used StrokeViewer’s vessel segmentation algorithm to segment the vessels in the CTA. The algorithm is a Residual 3D U-Net [27, 28].

#### Occlusion-downstream area selection

We used the hypoperfused area, determined at Tmax > 6 s, as the occlusion-downstream area. Tmax is a robust parameter for identifying hypoperfused tissue [29]. We use increased Tmax to represent the occlusion-downstream area as it captures tissue with delayed perfusion due to arterial blockage, making it a good proxy for the area downstream of an occlusion. The threshold Tmax > 6 s is a commonly used threshold for the definition of hypoperfusion across various CTP analysis software packages. Since typical CTP software removes vessels from analysis, we refined the downstream area to include vessels. We generated a mask from the estimated hypoperfused area and filled the empty spaces corresponding to vessels by applying morphological closing with a 3D ellipsoidal structuring element with semi-axes radii of ten voxels along the *x-* and *y*-axes, and one voxel along the *z*-axis. This approach makes vessels penetrating the downstream area part of the refined downstream area mask. We mirrored the refined downstream area mask in the midplane to obtain a similar area on the contralateral hemisphere.

#### Quantitative score

**Fig. 1:**
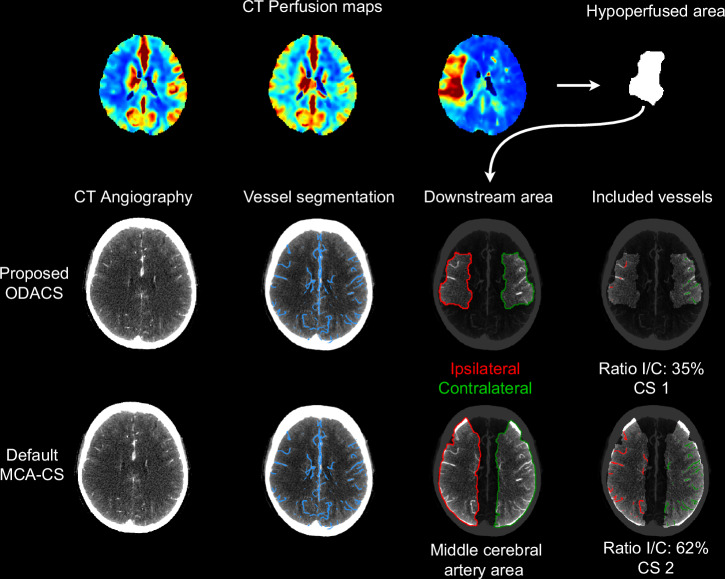
Example of our proposed method to obtain the ODACS, and the reference method to obtain MCA collateral score (MCA-CS). The top row shows the CTP maps and the hypoperfused volume. The vessel segmentation is shown in blue. Red and green regions show the ipsilateral and contralateral regions under consideration: the occlusion-downstream area from the hypoperfused area or the MCA territory. The last column shows the vessels specific to each region along with the quantitative score and categorized collateral score. All images are 10 mm maximum intensity projections

We calculated the total vessel volume within the occlusion-downstream area and its contralateral mirrored counterpart. We defined the ODACS as the ratio between the ipsilateral and contralateral vessel volume [5]. For visual representation of the results, we presented the identified vessels through a graphical user interface, highlighting the ipsilateral vessels (red) and the contralateral vessels (green). Figure 1 presents the workflow of the calculation of the proposed occlusion-downstream area CS.

#### Categorized score

Like most collateral scores (CS) used in clinical practice, we used thresholds to categorize the quantitative score. We based this on the four-point ordinal scale Tan score that categorizes collateral status, namely the absence of collaterals (CS 0, no filling of the occluded area), poor collaterals (CS 1, > 0% but ≤ 50% filling of the occluded area), moderate collaterals (CS 2, > 50% but < 100% filling of the occluded area), and good collaterals (CS 3, 100% filling of the occluded area) [5]. We adjusted the criteria to ensure that minor false positive segmentations did not mistakenly yield an incorrect category. For the CS 0 group, we, therefore, relaxed the criteria from no filling in the occluded area to less than 5% filling compared to the contralateral side. Similarly, for the CS 3 group, we relaxed the criteria to more than 95% filling compared to the contralateral side.

### Reference methods

#### MCA-territory collateral score

Since we hypothesized a reduction to lower collateral scores in the occlusion-downstream approach compared to the complete MCA-territory, we also calculated the scores throughout the MCA-territory. We used the Kaffenberger neuroanatomical CT-MRI brain atlas to create an MCA-territory mask [30, 31]. We defined the quantitative collateral score as the ratio of the vessel volumes between the ipsilateral and contralateral MCA territories. We refer to this score as the MCA-CS.

#### Visual occlusion-downstream area collateral score

To validate our automated ODACS method, we compared it against visual ODACS as assessed by two experienced neuroradiologists, R1 (C.M.) and R2 (B.E.), with 27 years and 17 years of experience, respectively. Following the definitions of the Tan score, both raters provided a reference standard by visually estimating the collateral scores for all available MR CLEAN-NO IV patients, utilizing the CTA and CTP perfusion maps, as well as the occlusion segment and side. To align better with the proposed continuous ODACS, we explored the use of an extended Tan score [32] for a more granular assessment, for one rater (R1). The extended Tan score subdivides CS 1 into CS 1a (> 0% but ≤ 25% filling of the occluded area) and CS 1b (> 25% but ≤ 50%), and CS 2 into CS 2a (> 50% but ≤ 75%) and CS 2b (> 75% but < 100%).

### Analysis

We carefully reviewed each case visually to ensure quality standards for midplane alignment, co-registration, and vessel segmentation. Patients not meeting these quality standards or patients without hypoperfusion were excluded from the analysis. We calculated the collateral scores across three groups of occlusion locations: proximal M1, distal M1, and M2. In our study, we defined the (post-bifurcation) M2 occlusions as distal. We analyzed the results for the three occlusion locations with Bland–Altman plots for the quantitative MCA-CS and ODACS, and with confusion matrices for categorized scores. We tested our hypothesis, a reduction to lower collateral scores when only the occlusion-downstream is considered compared to the MCA-territory, with a one-sided Wilcoxon Signed Rank test with confidence level 0.05 (python SciPy v1.12.0 [33]). We used the same analysis to assess if the hypoperfused volume was smaller than the MCA volume. Furthermore, we examined the proportion of patients who shifted from the clinically relevant dichotomized collateral score good (CS 2–3) to poor (CS 0–1) when using ODACS instead of MCA-CS. We analyzed inter-rater agreement and agreement between our method and the experienced raters with the quadratically weighted Cohen’s kappa statistic.

### Supplementary analysis

We conducted supplementary analyses to further validate the proposed methodology. These additional analyses focused on two key aspects: evaluating the performance of the vascular segmentation component in our method and assessing the sensitivity of our approach when using different CTP software packages. These analyses provide additional support for the robustness and reliability of the ODACS method and are presented in the Supplementary Materials.

## Results

**Fig. 2:**
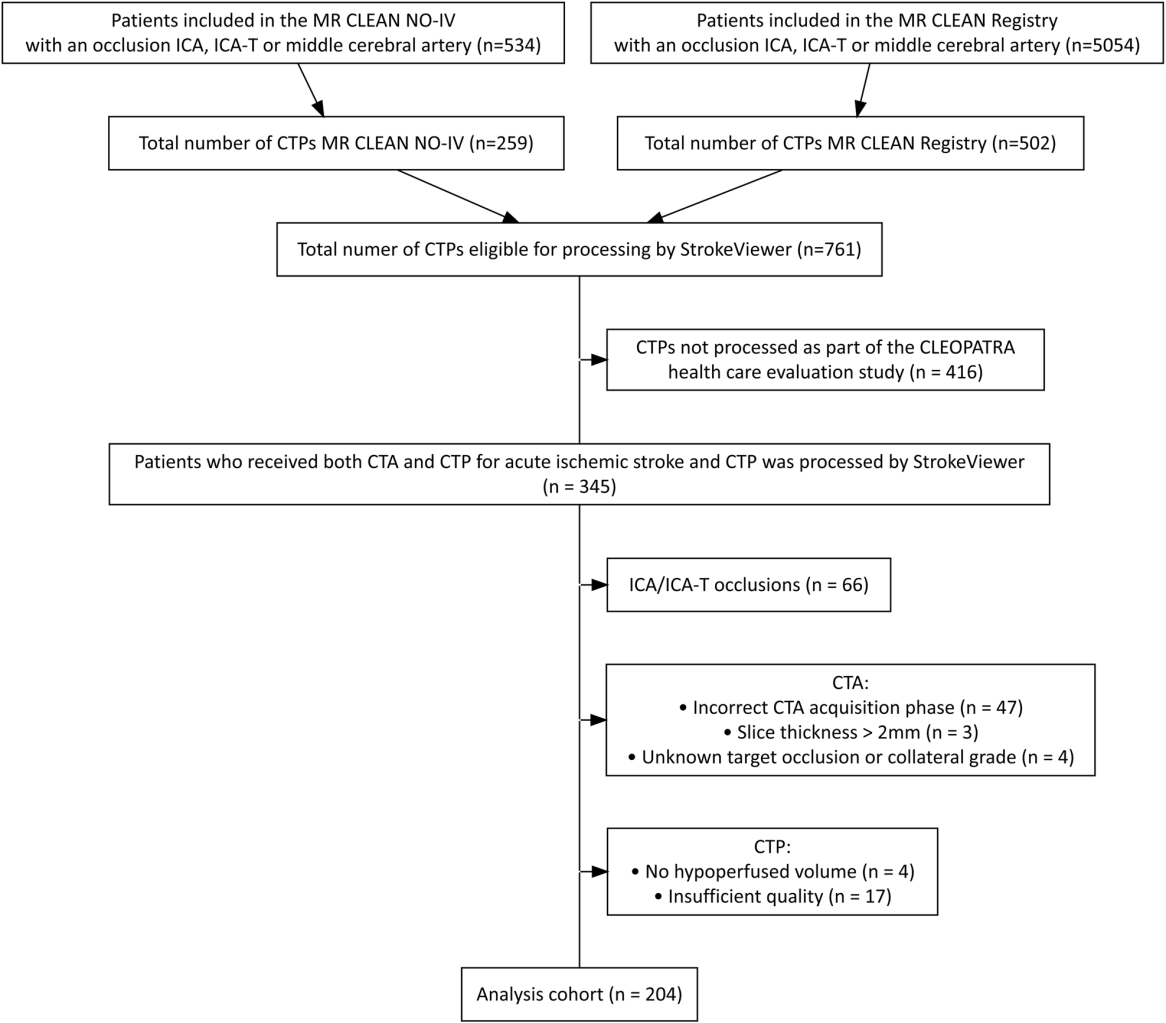
Flowchart to obtain our final study cohort

Of the 345 patients with baseline CTA, CTP images, and StrokeViewer results available, 204 were included (Fig. 2). Table 1 lists characteristics for the patients in our analysis with proximal M1 (*n* = 106), distal M1 (*n* = 65), and M2 (*n* = 33) occlusions. Figure 1 illustrates ODACS and MCA-based scoring for a patient with a proximal M1 occlusion. Even with proximal M1 occlusions, the downstream area can be smaller than the MCA-territory, leading to a different collateral score.

**Table 1:** Patient characteristics of the included population

	Included population (*n* = 204)
Patient characteristics	
Age (years)-median (IQR)	72 (63–80)
Male sex-*n* (%)	105 (51)
NIHSS score-median (IQR)	16 (12–20)
Intravenous thrombolysis-*n* (%)	142 (70)
Previous stroke-*n* (%)	43 (21)
Baseline imaging-NCCT	
ASPECTS noncontrast CT-median (IQR)	9 (8–10)
Baseline imaging-CTA	
Occluded segment-*n* (%)	
Proximal M1	106 (52)
Distal M1	65 (32)
M2	33 (16)
Times	
Onset to groin puncture (min)-median (IQR)	140 (111–190)
Dates	
Range study dates –mm/yyyy	05/2016 to 10/2020

*NIHSS* National Institutes of Health Stroke Scale

### Proximal M1 occlusions

**Fig. 3:**
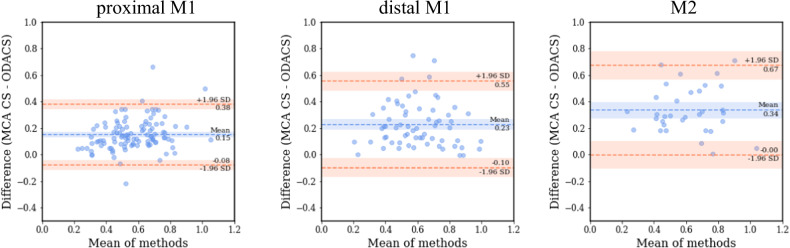
Bland–Altman plots for the MCA-CS and ODACS for proximal M1, distal M1, and M2 occlusions. The difference is calculated as MCA-CS–ODACS. The subfigures show a larger mean difference for more distal occlusion

**Fig. 4:**
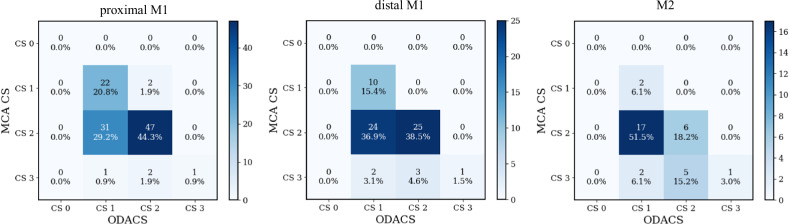
Confusion matrices for the categorized MCA-CS and ODACS. The subfigures for proximal M1, distal M1, and M2 occlusions indicate that 34 (proximal M1), 29 (distal M1), and 24 (M2) patients obtained a lower ODACS compared to MCA-CS

The MCA-CS was generally higher than the ODACS for proximal M1 occlusions. The Bland–Altman plot in Fig. 3 shows a mean of 0.15 for the difference MCA-CS–ODACS, suggesting a bias to higher collateral scores for the MCA-based approach. The confusion matrix in Fig. 4 shows that 34/106 (32%) patients obtained a lower ODACS compared to MCA-CS. The quantitative and categorized ODACS were significantly lower (*p* < 0.001) than the MCA-CS for proximal M1 occlusions. For two patients, the categorized ODACS was higher than the MCA-CS. In total, 32/106 (30%) patients shifted from good to poor collateral scores. The quantitative results in Table 2 indicate that the median (IQR) considered volume for the MCA-CS was 304 (295–309) mL, while the hypoperfused volume was 166 (122–206) mL.

**Table 2:** Median (IQR) values of the quantitative and categorical middle cerebral artery territory collateral score (MCA-CS) and occlusion- downstream area collateral score (ODACS), and the corresponding volumes of the ipsilateral MCA volume and hypoperfused volume. Additionally, the table lists the percentage of patients that shifted from the good collateral to the poor collateral group

Occlusion location	Quantitative ODACS	Quantitative MCA-CS	*p*-value
Proximal M1	0.49 (0.37–0.61)	0.63 (0.52–0.78)	< 0.001
Distal M1	0.46 (0.34–0.62)	0.69 (0.58–0.86)	< 0.001
M2	0.44 (0.32–0.56)	0.77 (0.61–0.92)	< 0.001

	Categorized ODACS	Categorized MCA-CS	*p*-value
Proximal M1	1 (1–2)	2 (2–2)	< 0.001
Distal M1	1 (1–2)	2 (2–2)	< 0.001
M2	1 (1–2)	2 (2–2)	< 0.001

	Ipsilateral hypoperfused volume	Ipsilateral MCA volume	*p*-value
Proximal M1	166 (122–206) mL	304 (295–309) mL	< 0.001
Distal M1	134 (100–183) mL	304 (300–310) mL	< 0.001
M2	86 (50–153) mL	308 (297–311) mL	< 0.001

	Shift from good to poor collaterals		
proximal M1	32/106 (30%)		
distal M1	26/65 (40%)		
M2	19/33 (58%)		

*MCA-CS* middle cerebral artery territory collateral score, *ODACS* occlusion-downstream area collateral score

#### Distal M1 and M2 occlusions

The MCA-CS was also higher than ODACS for distal M1 and M2 occlusions. The Bland–Altman plots in Fig. 3 show a mean difference MCA-CS–ODACS of 0.23 for distal M1 occlusions and 0.34 for M2 occlusions, implying a tendency toward a larger bias in the collateral score of more distal occlusions for MCA-CS. Furthermore, the spread in mean differences increases for more distal occlusions compared to the proximal M1 occlusions. Correspondingly, the confusion matrix in Fig. 4 shows that 29/65 (45%) distal M1 and 24/33 (73%) M2 patients obtained lower collateral scores when using ODACS. This is mirrored by the fact that the quantitative and categorized ODACS are significantly lower (*p* < 0.001) than the MCA-CS for distal M1 and M2 occlusions. The ODACS was never higher than the MCA-CS in these subsets. Of 26/65 (40%) distal M1 patients and 19/33 (58%) M2 patients shifted from the good collateral group to the poor collateral group. Table 2 indicates that for distal M1 patients, the median (IQR) volume under consideration for the MCA-CS was 304 (300–310) mL, while the hypoperfused volume was 136 (100–183) mL. For M2 patients, the considered volume for the MCA-CS was 308 (297–311) mL, while the hypoperfused volume was 86 (50–153) mL.

#### Visual ODACS

**Fig. 5:**
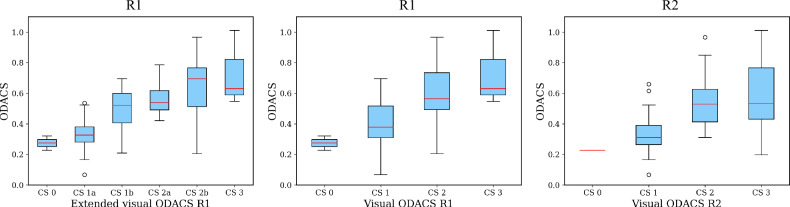
Box plots for the ODACS score vs the visual occlusion-downstream collateral scores from the two expert raters

Fig. 5 shows box plots for the quantitative ODACS and the visually scored ODACS. For patients with visually scored ODACS 0, our automated ODACS estimated higher collateral status. Alternatively, for patients with visual ODACS 3, the ODACS was lower than the visual score. The Cohen’s kappa statistic for the inter-rater agreement between R1 and R2 was κ = 0.45 (moderate agreement). The agreement between ODACS and R1 and R2 was κ = 0.51 (moderate agreement) and κ = 0.35 (fair agreement), respectively. The agreement between ODACS and R1 using the extended Tan scale was 0.55 (moderate agreement). R1 classified 9 patients as visual ODACS 3 and R2 classified 3 patients as visual ODACS 3. The experts agreed on 2 patients with visual ODACS 3.

## Discussion

In this study, we presented a novel quantitative assessment of collateral status in the downstream area of an occluded intracranial artery. For proximal M1, distal M1, as well as M2 occlusions, we found lower ODACS compared to using the whole MCA-territory (MCA-CS). The difference between ODACS and MCA-CS was greater for more distal occlusions. Additionally, we have shown that the agreement between ODACS with experts is on par with the interobserver agreement of the experts.

For proximal M1 occlusions, we expected the ODACS and MCA-CS to be more aligned. Our study, however, found that about one-third of patients with proximal M1 occlusions obtained a lower collateral category using the downstream method. Additionally, we found that the average ipsilateral volume of the downstream area of proximal M1 occlusions was approximately half of the entire MCA volume. This observation may be due to anatomical variations or watershed areas which are included in the entire MCA volume but not in the hypoperfused area, or limited coverage of the CTP. The ODACS may provide a patient-specific approach for the downstream area, however, it may underestimate the downstream area in cases of limited CTP coverage. The impact of this limitation needs further validation, given that CTP typically covers the greater portion of the MCA territory.

We used the hypoperfused area to define the downstream area. Alternatively, Boers et al calculated average occlusion territory maps from follow-up NCCT infarct core segmentations [34]. Using follow-up imaging might not be representative for the situation however, as underestimation could occur due to EVT, as well as overestimation due to infarct growth [35]. Moreover, Boers et al made no distinction between occlusions in proximal and distal M1 vessel segments or superior and inferior M2 trunks, and the probability maps of M2 and M1 territories were visually quite similar. While we could create an atlas or territory map for proximal occlusions, doing so for distal occlusions could prove difficult due to the considerable variations in vessel anatomy. Moreover, both visual and automatic occlusion localization have been shown to be challenging for distal occlusions [21, 36]. By considering the hypoperfused region, we obtained a patient-specific downstream area that inherently captures the vessel anatomy. The downstream areas of superior and inferior M2 trunks, for example, vary not only within patients but also across patients, and using the hypoperfused area as a downstream area surrogate captures these variations. Moreover, determining the downstream area is invariant to occlusion localization.

An argument against using the hypoperfused region as a surrogate for the downstream area might be that the downstream area of patients with rapid collateral supply (with Tmax < 6 s) could be underestimated. In this case, our method would not consider these collateral vessels when calculating the ODACS, and the collateral grade in the hypoperfused region might be underestimated. Future studies should investigate whether other definitions of the hypoperfused region (e.g., Tmax > 4 s) might improve the representation of the ‘true’ downstream region in patients.

The moderate inter-rater agreement between our expert readers highlights the importance of developing an automated assessment of downstream collateral status for distal occlusions. Our method primarily deviated from the raters in cases with minimal or no collateral circulation. In these instances, the ODACS tended to be higher than the threshold for the lowest collateral score category, possibly due to the segmentation of weakly attenuated vessels or noise leading to false positive vessel segmentations. This issue occurred for two patients. Furthermore, the discrepancy with higher visual ODACS might originate from an overestimation in the visual assessment. This is particularly evident considering that R1 and R2 decided on visual ODACS 3 for only two patients, highlighting the challenging nature of this task.

The ODACS provides a more accurate assessment of collateral status in distal occlusions compared to the MCA-CS, which overestimates collateral capacity. Clinicians using the MCA-CS might expect good treatment effects based on an overestimated collateral score, whereas the ODACS could offer a more representative evaluation. This refinement in collateral assessment has potential implications for patient selection and management in acute stroke care, particularly for distal occlusions. By more accurately categorizing patients into good or poor collaterals, the ODACS may support more informed decision-making in treatment strategies, potentially improving patient outcomes.

Downstream collateral scoring may, for example, prove useful to evaluate the effects of collaterals on potential treatment benefits in EVT trials. The recent MR CLEAN LATE Trial identified a patient group who benefited from endovascular therapy in the late time window, based on the presence of collaterals in the MCA-territory compared to the unaffected side [37]. Notably, subgroup analyses revealed that patients with poor collateral status (< 50% compared to the unaffected side) had greater treatment effects compared to patients with good collateral status. An explanation for this could be the overrepresentation of patients with distal occlusions in the group with good collaterals. This may be due to the MCA-territory-based scoring method, leading to higher collateral scores for distal occlusions. Therefore, further research into downstream collateral assessment is needed, particularly focusing on its clinical relevance for the treatment effect of EVT. Such research could also provide insights into the potential advantages of occlusion downstream collateral assessment for guiding treatment decisions.

Our study has limitations. First, our method requires CTP imaging, which was not consistently available in our dataset, particularly in the MR CLEAN Registry, limiting our ability to evaluate ODACS on a larger proportion of patients. However, CTP is currently widely available. Second, our dataset contains only a limited proportion of more distal occlusions, with distal M1 and M2 occlusions accounting for 32% and 16% of cases, respectively. The limited proportion of more distal occlusions in our dataset directly results from the fact that relatively few patients with distal M1 and M2 occlusions have been treated with endovascular therapy to date. Third, our method uses the hypoperfused region obtained from CTP software, and there is variation between different vendors’ CTP software [38, 39]. Our evaluation study, however, shows that ODACS is robust to using different vendors’ CTP software. Another limitation is that while our post-processing approach smoothens the border of the hypoperfused region to include vessels, this could have affected the score calculation. Moreover, variation in image quality (e.g., noise) might have influenced the vessel segmentation and CTP outcomes quality. Another limitation is that we excluded patients with venous flow, yet minor venous filling cannot be entirely excluded. Early timed CTA may underestimate the collateral score, which could have more impact on distal occlusions. Alternatively, late-timed CTA may overestimate the collaterals, as there is more venous filling. Using thin-slice CTP or multi-phase CTA source data for segmentation of the vessels at peak arterial phase could mitigate these issues. For our study, however, such data were not available. Lastly, our study is limited by the use of only two raters for the visual ODACS scoring, which may restrict the generalizability of our inter-rater agreement findings.

In conclusion, we presented a novel approach for collateral assessment for more distal vessel occlusions. The ODACS collateral score resulted in lower scores than the MCA-based method, leading to a shift in classification from good to poor collateral status, particularly for more distal occlusions. ODACS demonstrates fair to moderate agreement with expert raters, who themselves show moderate agreement with each other.

## Supplementary information

ELECTRONIC SUPPLEMENTARY MATERIALdocx

## Abbreviations

CS: Collateral score
CT: Computed tomography
CTA: Computed tomography angiography
CTP: Computed tomography perfusion
EVT: Endovascular treatment
MCA: Middle cerebral artery
ODACS: Occlusion-downstream area collateral score
