# Supplementary material for: Automated collateral assessment restricted to the hypoperfused area for distal vessel occlusions in ischemic stroke
Source: Eur Radiol. 2025 Apr 14;35(10):6127–39. doi: 10.1007/s00330-025-11442-2 (PMC12417235; doi:10.1007/s00330-025-11442-2)
Supplement: Supplementary file 1 — ELECTRONIC SUPPLEMENTARY MATERIALdocx [file 330_2025_11442_MOESM1_ESM.docx]

# Supplementary materials

To validate our proposed methodology we performed additional analyses to assess the segmentation performance component of our method and the sensitivity of our method to different CT perfusion software packages.

**Dataset**

Data from 25 patients in the MR CLEAN-NO IV trial with ICA-T (4), proximal M1 (15), and distal M1 (6) occlusions were included. All patients had NCCT, CTA, and CTP datasets available. The CTP data was processed with three software packages: StrokeViewer (Nicolab), Syngo.via CT Neuro Perfusion (VB40, Siemens Healthineers), and Vitrea Bayesian CT Brain Perfusion 2D (version 7.14; Vital Images). All CTAs were acquired in the arterial phase. Patients had right hemisphere occlusions in 42% of cases, and left hemisphere occlusions otherwise. This dataset of 25 patients was used for both assessing the segmentation performance and evaluating the sensitivity of our method to different CT perfusion software packages,

**Experiment 1: vessel segmentation performance**

Two experiments were conducted to assess the performance of the segmentation model. In the first experiment, 15 markers per patient were placed across horizontal M1, vertical M2, and horizontal M3 segments in the healthy hemisphere (5 per segment). Markers that intersected with the generated vessel segmentation were considered successfully retrieved. The marker retrieval rate [1] was calculated as the percentage of markers retrieved for each segment. For the second performance evaluation, an experienced rater (BE) qualitatively scored the vascular segmentation quality following the criteria in Table A1. The vessel segmentation was presented as an overlay on the original CTA, and the segmentations could be toggled on and off.

The marker retrieval rates were 90% for markers in M1 segments, 100% for markers in M2 segments, and 99% for markers in M3 segments. Of all patients, the rater scored 13/25 (52%) of the segmentations as 'excellent' and 9/25 (36%) as 'good'. The remaining segmentations were classified as 'fair' (4%) or 'poor' (8%). None of the segmentations were classified as 'very poor'. The most frequent cause for low-quality segmentations was the under-segmentation of larger vessels.

**Experiment 2: ODACS robustness to different CT perfusion analysis software packages**

We assessed the effect of variation in CT perfusion analysis software packages on the ODACS. The ODACS for 25 patients were determined using the hypoperfused region maps from three software packages. Figure A1 shows the ODACS for all patients for the three software packages in the left panel, and the average, minimum, and maximum ODACS for each patient in the right panel. There is limited variation in the ODACS among software packages. The median (IQR) difference in ODACS between Syngo.via and StrokeViewer (Syngo.via - StrokeViewer) is 0.05(0.02 - 0.10). For Vitrea and StrokeViewer, the median(IQR) difference (Vitrea - StrokeViewer) in ODACS is 0.03(-0.01 - 0.06).

For a single patient, the ODACS utilizing Vitrea perfusion maps is significantly higher than for the other software packages. This is because Vitrea selects the incorrect hemisphere as the affected hemisphere.

The findings indicate that the choice of software has only a limited impact on the ODACS, suggesting that our method is robust across different CTP post-processing software packages.

Table A1:

Criteria for qualitative scoring of vessel segmentation quality. Scores range from 'Very poor' to 'Excellent' based on the accuracy and completeness of vessel segmentation.

| **Score** | **Criterium** |
| --- | --- |
| *Very poor* | Most vessels are missed or incorrectly segmented |
| *Poor* | Vessel boundaries are largely inaccurate; many noticeable false positives or false negatives |
| *Fair* | Some inaccuracies in vessel boundaries; noticeable false positives or false negatives |
| *Good* | Most vessels are accurately segmented; Minor inaccuracies in some vessel boundaries |
| *Excellent* | Complete and accurate segmentation of all visible vessels |

**Figure A1:**

Comparison of ODACS across different CT perfusion software packages. Left panel: ODACS for 25 patients calculated using StrokeViewer, Syngo.via, and Vitrea CTP analysis software packages. Right panel: Average ODACS with minimum and maximum values for each patient across the three software packages. Note the outlier patient where Vitrea incorrectly selected the unaffected hemisphere, resulting in a significantly higher ODACS. Our categorized collateral score (CS) categorizes collateral status into four groups: CS 0 (<5% filling), CS 1 (5-50% filling), CS 2 (>50% to <95% filling), and CS 3 (≥95% filling) of the occluded area compared to the contralateral side.


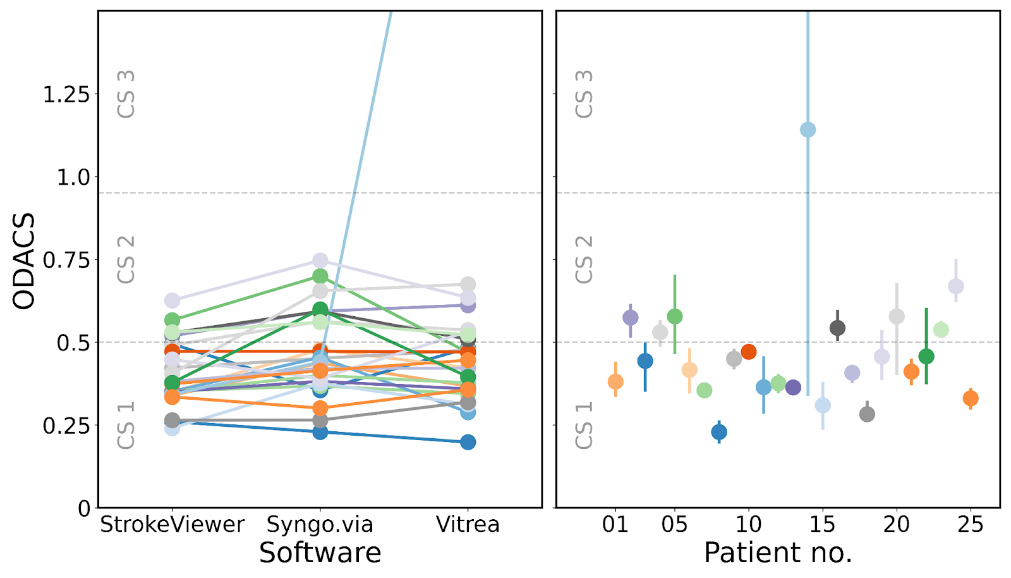


# References

1. Herten RLM Van, van Harten L, Planken N, Isgum I (2024) Generative Adversarial Networks for Coronary CT Angiography Acquisition Protocol Correction with Explicit Attenuation Constraints. In: Oguz I, Noble J, Li X, et al (eds) Medical Imaging with Deep Learning. PMLR, pp 1288–1303
